# Phenotype-Genotype Correlation in Wilson Disease in a Large Lebanese Family: Association of c.2299insC with Hepatic and of p. Ala1003Thr with Neurologic Phenotype

**DOI:** 10.1371/journal.pone.0109727

**Published:** 2014-11-12

**Authors:** Julnar Usta, Antonios Wehbeh, Khaled Rida, Omar El-Rifai, Theresa Alicia Estiphan, Tamar Majarian, Kassem Barada

**Affiliations:** 1 Department of Biochemistry and Molecular Genetics; Faculty of Medicine, American University of Beirut, Beirut, Lebanon; 2 Faculty of Medicine, American University of Beirut Medical Center, Beirut, Lebanon; 3 Division of Gastroenterology, Department of Internal Medicine, American University of Beirut Medical Center, Faculty of Medicine, Beirut, Lebanon; University of Saskatchewan, Canada

## Abstract

Genotype phenotype correlations in Wilson disease (WD) are best established in homozygous patients or in compound heterozygous patients carrying the same set of mutations. We determined the clinical phenotype of patients with WD carrying the c.2298_2299insC in Exon 8 (c.2299insC) or the p. Ala1003Thr missense substitution in Exon 13 mutations in the homozygous or compound heterozygous state. We investigated 76 members of a single large Lebanese family. Their genotypes were determined, and clinical assessments were carried out for affected subjects. We also performed a literature search retrieving the phenotypes of patients carrying the same mutations of our patients in the homozygous or compound heterozygous state. There were 7 consanguineous marriages in this family and the prevalence of WD was 8.9% and of carriers of *ATP7B* mutation 44.7%. WD was confirmed in 9 out of 76 subjects. All 9 had the c.2299insC mutation, 5 homozygous and 4-compound heterozygous with p. Ala1003Thr. Six of our patients had hepatic, 2 had neurologic and 1 had asymptomatic phenotype. Based on our data and a literature review, clear phenotypes were reported for 38 patients worldwide carrying the c.2299insC mutation. About 53% of those have hepatic and 29% have neurologic phenotype. Furthermore, there were 10 compound heterozygous patients carrying the p. Ala1003Thr mutation. Among those, 80% having c.2299insC as the second mutation had hepatic phenotype, and all others had neurologic phenotype. We hereby report an association between the c.2299insC mutation and hepatic phenotype and between the p. Ala1003Thr mutation and neurologic phenotype.

## Introduction

Wilson disease (WD; MIM# 277900) is an autosomal recessive, copper transport disorder characterized by extensive phenotypic diversity [1], [2]. Patients may present at any age with hepatic, neurologic, or mixed symptoms. Yet some may be asymptomatic [3]. WD is due to a defective *ATP7B* gene (OMIM*606882; Ref seq accession #: NM_000053.3); that is located on chromosome 13 (Gene map locus: 13q 14.3–921.1) that encodes a copper transporting p-type ATPase [4], [5]. More than 500 mutations have been identified so far, and ongoing efforts to associate these with disease phenotypes have been inconclusive and controversial [6]. Inability to establish genotype-phenotype correlations may be attributed to the large number of mutations that occur in only few families, and to the heterogeneity of the clinical presentation of WD patients even within members of the same family. The fact that the majority of patients are compound heterozygote, having a different mutation on each allele, makes it hard to relate a phenotype with one mutant allele. Furthermore occupational exposure to copper has been shown to cause genomic alterations and DNA damage [7]. This in combination with epigenetic modulators and environmental factors may play a role in the phenotypic heterogeneity of WD patients [8]. These difficulties may be partially overcome by studying WD in homozygous patients [9].

Specific mutations in the ATP7B gene are more frequent in populations where consanguineous marriages are prevalent. In Lebanon, consanguinity has a prevalence of 35.5%, increasing the probability of homozygozity for autosomal recessive diseases [10].

We have previously reported on multiple Lebanese families with members affected with WD. We found an association between the homozygous missense mutation p. Gly691Arg (Exon-7) with early and severe hepatic disease [11]. In addition an association between liver disease and homozygous mutations in the conserved ATP hinge region (Exon-18: p. Asn1270Ser and p. Pro1273Leu) of the *ATP7B* gene [9] was suggested. In a recent study we reported that patients with homozygous missense mutations, other than p. His1069Gln, are more likely to have a hepatic phenotype, liver failure and present at a younger age [12].

In this paper, we report on the phenotype and genotype of 9 patients with WD who belong to a single large family with extensive consanguinity. Five of our patients were homozygous for c. 2299insC (Exon-8), and four were compound heterozygous for both the c. 2299insC (Exon -8) and the missense mutation p. Ala1003Thr (Exon-13). Both mutations were previously reported as disease causing mutations [8], [13], [14]. A literature search retrieving reported phenotypes of all patients carrying either one or both of these two mutations was performed and compared to our own. We hereby suggest that the c. 2299insC favors a hepatic phenotype while the p. Ala1003Thr favors a neurologic phenotype.

## Materials and Methods

### Patients/Subjects

A total of 235 individuals distributed over 6 generations in a single extended Lebanese family, the S-family, were identified. This family came to our attention after some of its members were diagnosed with WD at the American University of Beirut Medical Center (AUBMC). Seventy six subjects (S1- S76) belonging to the S-family were enrolled in the study. Whenever an index case of WD was identified, a full clinical and genetic evaluation of that patient was conducted at AUBMC, while genotypic analysis was performed on the patient as well as all his/her family members. Out of the 76 subjects, 9 were found to have WD. Patient evaluation consisted of a full history, complete physical and neurological examination, ophthalmologic slit-lamp examination for Kayser-Fleischer (KF) rings, abdominal ultrasound, and standard biochemical tests including liver function tests, serum ceruloplasmin, serum copper and 24 hr urinary copper levels. Ceruloplasmin level was determined using immuno-nephlometric method (BN ProSpec analyzer system, from Siemen. Serum and urine copper levels were performed in a reference Laboratory (CERBA, France) as service, using Atomic absorption and ICP-MS, respectively. Five patients had brain MRI and two had EEG done as part of their evaluation.

DNA screening for exons bearing mutations or single nucleotide polymorphisms (SNPs) was performed in all recruited subjects. Cases were labeled as hepatic, neurologic, or asymptomatic following Ferenci's classification [15].

Detailed information was obtained from available family members in order to construct an accurate pedigree representing the 235 individuals of the S-family. Twelve members of the S- family died of WD according to information provided by their family members. Data pertaining to the clinical and genetic profiles of these deceased patients could not be obtained.

### Ethics Statement

Written informed consent for participation in this study was obtained from all subjects or their guardians. The study protocol #: BioCh. JU.01 was approved by the International Review Board and the Research Committee at the American University of Beirut- Medical Center.

### Mutation Analysis

#### Materials and Reagents

Various reagents used in this study were purchased from the following suppliers: IQ supermix, and Agarose from Bio-Rad; DNA purification Kit using Nucleospin Extract II columns from Machery Nagel, Germany (Cat no. 740609-50); T4 Kinase Polynucleotide from Invitrogen; γ-^32^P- ATP from Amersham (3000 Ci/mmol); SSDNA from Sigma Aldrich cat# D7656; Sephadex columns from GE Healthcare (Microspin G-25 column illustra 27-5325-01); and T4-Kinase from Invitrogen, Life Technology.

DNA concentration was quantified using the Gene Quant Spectrophotometer. PCR was performed using My Cycler Thermal Cycler from Bio-Rad; Membrane hybridization and crosslinking were carried out using ProBlot 12 Hybridization Oven Labnet, (31 Mayfield Avenue Edison, NJ, 08837 USA), and Spectrolinker UV Crosslinkers from Krackeler Scientific, (Inc. PO Box 1849 Albany, NY 12201-1849), respectively. Sequencing of purified DNA was carried out at the University of Saint Joseph, Department of Molecular Biology and Genetics using the Avant Genetic Analyzer (ABI 3130) machine. The sequencing reaction and subsequent purification steps were as described before [9].

#### Genotypic Analysis

Blood samples were collected in EDTA containing tubes, from 9 WD patients of the S-family and related members for DNA isolation. Blood samples were collected in EDTA containing tubes from 9 WD patients of the S-family and related members for DNA isolation. In brief Red Cell Lysis Buffer (RCLB composed of: 7.7 g ammonium chloride and 0.1 g potassium bicarbonate were dissolved in 1l water) was added to blood sample (2v/1v), incubated at 37°C for 12 min, and centrifuged (4000rpm,2 min). The resultant pellet was re-suspended in RCLB (2 ml) followed by the consecutive addition of: white cell lysis buffer (1.8 ml, WCLB composed of: 1.2 g TRIS, 1.68 g EDTA, 23.4 g sodium chloride dissolved in 1l water), SDS (24 µl of 10%) and proteinase–K (18 µl of 20 mg/ml). The suspension was incubated for 2 hrs at 55°C and proteins were salted out by adding 600 µl of 5 mM NaCl, followed by centrifugation at 4000 rpm for 15 min. The supernatant was transferred to a clean tube where an equal volume of ethanol (99%) was added to precipitate DNA. The flocculent DNA was transferred into 1.5 ml eppendorf tube, washed with 70% ethanol (200 µl), and centrifuged at maximum speed for 1min. The supernatant was then discarded. The pellet was allowed to air-dry and was then finally suspended in 200 µl TE buffer. DNA concentration was determined using Gene Quant Spectrophotometer at λ = 260 nm, and samples were stored at −20°C.

#### Polymerase Chain Reaction (PCR)

Amplification of Exons 2–21 of WD gene were carried on all samples using My Cycler, (BIO-RAD). Primers flanking the exons' boundaries were designed (will be provided upon request) according to Petrukhin et al [16] with minor changes using primer 3 software (http://frodo.wi.mit.edu/cgibin/primer3/primer3_www.cgi). The PCR reaction mixture contained in a final volume of 50 µl: 25 µl IQ supermix (BIORAD), 22 µl H_2_O, 1 µl of each of the primers (forward, reverse) each at a concentration of 3.4 pmoles/µl, and 1 µl DNA (250 ng). Two PCR programs were used in amplifying the exons:

**Program -1** involved activation of Taq polymerase (94°C, 2 min), 38 cycles of denaturation (94°C, 30 sec), annealing (59°C, 30 sec), Extension (72°C, 40 sec) and a final extension (72°C, 7 min) followed by hold step. This program was used to amplify exons: 2, 4–10, 15 and 17.

**Program- 2** was similar to program 1, except for the annealing temperature it was 61°C. This program was used to amplify exons: 3, 11–14, 16, and 18–21. Amplified PCR products were then separated on 2% agarose gel, compared to DNA ladder of Molecular weight standards. DNA band corresponding to appropriate size were cut and purified using gel-extraction kits, Nucleospin Extract II columns (Machery Nagel), following Manufacturer's instructions. Sequencing of amplified exons was performed on WD patients as detailed in [9], [12], compared to published normal sequences in the various databanks either Blast at National Center For Biotechnology Information (http://www.ncbi.nih.gov/Blast) or Blat at University of California Santa Cruz, Genome Bioinformatic site (http://www.genome.ucsc.edu/cgi-bin/hgBlat).

Genotypic screening of all other subjects was performed using DOT blot analysis following standard protocols [17]. Random selection of the amplified ATP7B exons: 8,10,12,13, and 16 from subjects (including WD patients) screened by Dot Blot, were sequenced to verify the Dot blot results.

### Dot Blot Analysis

#### a. Labeling of probes

Normal and mutant probes were designed to include nucleotide base changes (mutations, SNPs) identified in patients. Probes (Table S1) were labeled using gamma ^32^P-ATP as per instruction of the T4 kinase polynucleotide supplier. The labeled ^32^P -probes were then denatured and centrifuged onto sephadex G-25 columns for 1min at 2400 rpm, where samples of the eluted probe were counted using liquid scintillator.

#### b. Membrane Preparation and Hybridization

Amplified PCR products were diluted to 0.4 ng/µl with 0.4 M NaOH −10 mM EDTA, denatured by heating (10 min at 100°C), cooled & loaded on positively charged nitrocellulose membrane using Bio Blot machine (Bio-Rad), and washed consecutively with NaOH (100 µl, 0.4 M) followed by 2 X SSC (prepared as described in Current Protocols in Molecular Biology [17]). Briefly, the membrane was allowed to dry at room temperature and was then cross linked for 30 seconds using UV cross linker. Following pre-hybridization of membranes with aqueous prehybridization (APH) solution and denatured Salmon Sperm DNA (SSDNA) for 3hrs at 60°C, cross linked membranes were hybridized for 3–4 hours with labeled probes (10^6^ cpm/300 ng) (Exons: 8,10,12,13, 16 at 45°C, 42°C, 30°C, 53°C, 43°C respectively), and were then washed at different stringencies (Table 1), dried, wrapped and exposed onto X-Ray films for 24–72 hours.

**Table 1:** Summary of the washing stringency conditions optimized for the different normal and mutant probes.

Hybridization					
15ml APH + labeled probes for 3–4hours at specific annealing temperature					
Washings: Volume ml/Time min/Temperature °C					
	Exon8	Exon10	Exon12	Exon13	Exon16
2XSSC-0.1%SDS	15/10/25	15/10/25	20/30/25	15/10/25	25/25/25
2XSSC-0.1%SDS	15/10/25	15/10/25	20/25/25	15/10/25	25/20/25
0.2XSSC- 0.1%SDS	15/20/25	15/15/25	20/25/34	20/20/25	20/20/25
0.2XSSC- 0.1%SDS	15/20/57	-	-	20/30/61	-
0.1XSSC-0.1%SDS	-	15/20/50	-	-	20/30/49

Radioactive ^32^P labeled normal and mutant probes of Exons: 8, 10, 12, 13, &16 were hybridized with amplified denatured DNA loaded on positively charged membrane. Membranes were then washed under different stringency conditions with SSC- SDS. The abbreviation SSC-SDS stands for: Sodium chloride: Tri-Sodium Citrate (3 M: 0.3 M, pH 7) - Sodium Dodecyl Sulfate used at the indicated volume in ml, time in minutes and temperature °C.

### Literature Review

We conducted a comprehensive literature search of PubMed and Medline for all articles published between 1993 and 2014 using the following index terms: Wilson disease and, mutation, genotype, phenotype. We also reviewed the articles mentioned in the University of Alberta database (Wilson Disease Mutation Database: http://www.wilsondisease.med.ualberta.ca/database.asp). We included all articles that clearly stated the phenotype of patients homozygous or heterozygous for either one of these 2 mutations: c.2299 insC in Exon-8 and p. Ala1003Thr substitution in Exon-13 of the *ATP7B* gene.

## Results

### Clinical features

**Figure 1:**
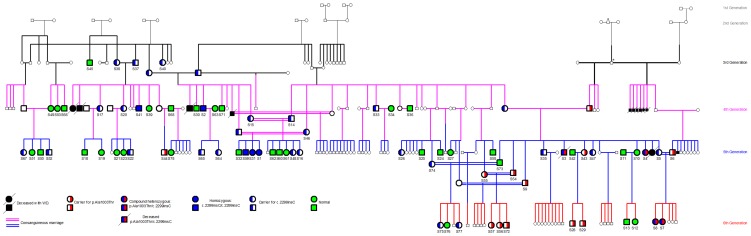
Pedigree of the S-family. The pedigree shows the 235 members of the S-family. The 76 members recruited in our study are highlighted. Nine members (S1, S2, S3, S4, S7, S8, S31, S41, S59) have WD. Generations were referred to by different colors: Gray for the 1^st^ and 2^nd^; Black for the 3^rd^; Pink for the 4^th^; Blue for the 5^th^ and Red for the 6^th^. * Refers to 2 men who were each married to 2 women.

A pedigree of the S-family is presented Fig.1 showing the 235 members, and including all affected individuals and their immediate families. There were 7 consanguinous marriages in this family. Out of the 76 individuals enrolled in this study, 9 (11.8%) were diagnosed with WD (5 females & 4 males), and those are coded as: (S1, S2, S3, S4, S7, S8, S31, S41, and S59). Seven of the 9 patients belonged to 3 nuclear families, these are: (S1, S31, S59), (S7, S8) and (S3, S4). Six patients had a hepatic phenotype, two had neurologic phenotype, and one was completely asymptomatic and diagnosed by screening. Twelve out of the remaining 159 members had passed away because of WD according to their family members. Their clinical characteristics and genotype could not be determined. The prevalence of WD in the S-family, taking into account both the alive and deceased individuals, is 8.9% (21/235).

Eight patients had decreased serum ceruloplasmin level, while patient S41 had a normal level. The 24-hour urinary copper level was markedly elevated in 8 patients (average of 695.1 µg/24 hr) but was normal in one patient (S59) who was diagnosed by screening at the age of 1 year. KF rings were present in 6 affected subjects.

The clinical profiles of affected individuals are summarized in Table 2. The average age at diagnosis was **11.2** years, while the mean age of patients with hepatic and neurologic manifestation were 9.7 and 14 years, respectively.

**Table 2:** Clinical, biochemical and genetic profile of WD patients in the S family.

	S1	S2	S3	S4	S7	S8	S31	S41	S59	
*Year of birth*	1993	1973	1981	1983	1993	1989	1997	1980	2007	
*Age (y) of symptoms onset*	-	12	15	13	12	-	-	16	-	
*Age (y) at diagnosis*	5 by screening^a^	12	16	14	12	15 by screening^a^	10 by screening^a^	16	1 by screening^a^	
*Clinical findings*										
GI manifestations	Asymptomatic transaminitis, hepatomegaly	Absent ^b^	Cirrhosis	Cirrhosis	Cirrhosis	Absent	Asymptomatic transaminitis, hepatomegaly	Absent	Asymptomatic fatty liver	
									
*Neurological*	Absent	Slurred speech, ataxia, tremors	Absent	Absent	Absent	Absent	Absent	Choreo-athetosis, tremors, rigidity	Absent	
									
Other	Absent	Arthralgia	Absent	Wilson's arthropathy	Absent	Absent	Absent	Absent	Absent	
									
MRI of Brain	Normal	Increased signal intensity in the basal ganglia & brain stem	—	Mild increase in signal intensity in the frontal lobes	Increase in signal intensity involving globus pallidus	Normal	—	—	—	
Kayser-Fleischer ring	Absent	Present	Present	Present	Present	Present	Absent	Present	Absent	
*Laboratory findings*										
Serum ceruloplasmin g/L	<0.04	0.072	0.096	0.096	0.17	0.12	0.03	0.423^b^	<0.019	
Serum Cu(µg/dL)	15	23	58	75	45	41	9	133.35	<5	
24h urine Cu (µg)	99	512	775	590	645	487	152.8	230°C	10	
ALT, AST (IU/mL)	289, 167	17, 13	30, 41	30, 35	29, 45	45, 41	105, 51	3, 20	44, 37	
Bilirubin T/D (mg/dl)	0.3/0.1	0.4/0.2	0.5/0.2	0.4/0.1	0.7/0.3	0.7/0.2	0.2/0.1	1/0.4	0.3/0.1	
Albumin (g/L)	50	43	35	43	37	40	45	45	47	
INR	1	1.3	1.3	1.9	1.2	1.1	—	1	1.1	
									
ATP7B Mutations*	2299insC/2299insC	2299insC/2299insC	2299insC/p. Ala1003Thr	2299insC/p. Ala1003Thr	2299insC/p. Ala1003Thr	2299insC/p. Ala1003Thr	2299insC/2299insC	2299insC/2299insC		2299insC/2299insC

a Screening refers to genetic screening of the ATP7B gene exons' by PCR followed by sequencing; **^b^**Few years later developed liver cirrhosis and portal hypertension;

**Normal ranges of**: Serum ceruloplasmin: 0.15–0.60 g/; Serum Cu: 70–150 µg/dL; 24h urine Cu: 15–50 µg/24h; Bilirubin T/D: 0.2–1.2/0–0.5; ^b^level determined after 7 years of treatment; ^c^Urinary Cu level while S41was on penicillamine; * c.2298_2299insC is referred to as 2299insC. DNA Mutation numbering is based on cDNA numbering where nucleotide +1 as the A of the ATG translation initiation codon, in the reference sequence # NM_000053 with the initiation codon aa codon +1.

Among the 6 patients with hepatic disease, 3 patients (S3, S4, and S7) had liver cirrhosis with portal hypertension. Patient (S3) presented with lethargy and gingival bleeding and subsequent endoscopy showed grade-3 esophageal varices. CT scan of the abdomen showed marked splenomegaly and a nodular liver consistent with cirrhosis. Patient (S4) had coarse hepatomegaly, splenomegaly, as well as a dilated portal vein by abdominal ultrasound, in addition to a prolonged INR ranging between 1.2–1.4, as well as arthropathy. Her brain MRI showed mild increase in signal intensity in the frontal lobes while her EEG was normal.

Patient (S7) presented with jaundice, increased abdominal girth, pitting edema, bleeding tendency along with generalized weakness. She had no neurologic symptoms and her neurologic exam was normal. Her brain MRI showed bilateral ill-defined symmetrical areas of increased signal intensity involving Globus Pallidus and posterior limbs of internal capsule. In addition her abdominal ultrasound revealed changes of chronic liver disease as well as portal hypertension.

Patients (S1, S31) were found to have hepatomegaly by abdominal ultrasound and asymptomatic transaminitis upon diagnosis, with their alanine aminotransferase levels being elevated to 118 U/L and 105 U/L respectively. S1 had a normal Brain MRI and an EEG showing rare sharp waves in the right parietal area. Patient (S59) had hepatic involvement confirmed by ultra-sonography showing fatty liver.

Two patients (S2, S41) presented with pure neurologic manifestations of WD at 12 and 16 years of age, respectively. The first presented with tremors, ataxia, slurred speech, in addition to arthropathy, while the second developed tremors, rigidity, and abnormal gait. Brain MRI of the former showed increased signal intensity in the basal ganglia and brain stem with involvement of brachium pontis bilaterally.

One patient (S8) who was asymptomatic had normal liver function tests, abdominal imaging, and brain MRI. The remaining 67 enrolled subjects were unaffected.

### Mutation Analysis

**Figure 2:**
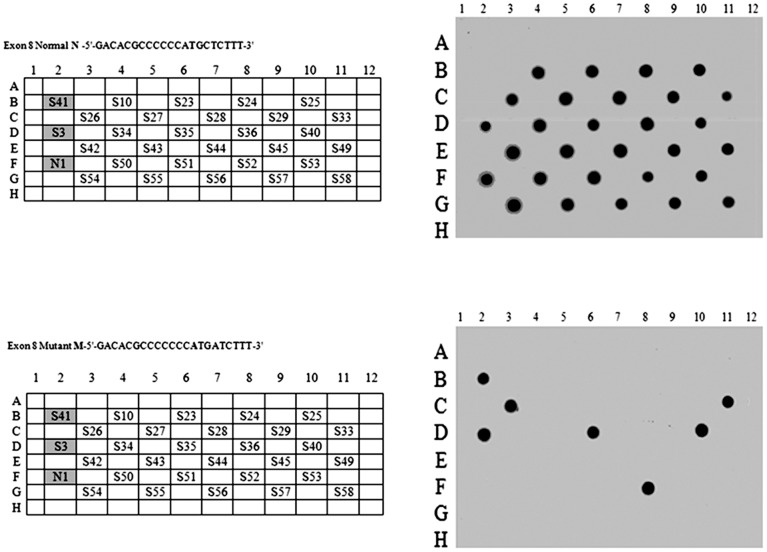
Dot blot image. A representative dot blot image of a membrane hybridized with normal and mutant probes of the identified exon 8 mutation. A dark spot resulting from hybridization with: Normal probe only, indicates a normal subject; Mutant probe only, indicates a homozygous subject; both normal and mutant probes indicate a carrier or heterozygous subject.

The spectrum of mutations in the *ATP7B* gene of the S family was determined by sequencing exons 2–21 in WD patients (9/76) of the S family. DNA mutation numbering is based on cDNA. Genotypic findings of disease causing mutations are listed in Table 2. Five patients (S1, S2, S31, S41, and S59) were homozygous for a frame shift mutation (c. 2299insC/c. 2299insC, Exon-8/8). Four patients, (S3, S4, S7 and S8) were compound heterozygous for the frame shift in exon- 8, and missense mutation (c.3007G>A substitution) in exon-13: (c. 2299insC/p. Ala1003Thr). In addition all affected patients were simultaneously homozygous for 2 SNPs in Exon-16 (p. Val1140Ala) and Exon-12 (p. Arg 952 Lys). A SNP in Exon-10 (p. Lys832Arg) was also identified in the homozygous state in patients S1, S2, S31, S41, and S59; in the heterozygous state in S7 and S8; while it was normal in S3 and S4. Patients had normal sequences in all remaining exons: 2–7, 9, 11, 14, 15, 17, 18, 19, 20, and 21. Further screening of related family members using dot blot (Fig. 2) identified a high rate of mutant allele carriers (44.7%) distributed as: 23/76 for the c. 2299insC (30.2%) and 11/76 for p. Ala1003Thr (14.5%). Random selection of the amplified ATP7B exons: 8, 10, 12, 13, and 16 from subjects screened by Dot Blot and including WD patients, were sequenced. Out of the 76 subjects a total of: 29 subjects for Exon-8; 19 subjects for Exon-10; 14 subjects for Exon-12; 23subjects for Exon-13, and 17 subjects for Exon-16 were sequenced confirming our Dot Blot findings.

### Literature review findings

A total of 17 articles were included based on the following criteria: 1) the patient carries one of the aforementioned mutations in the homozygous or heterozygous state, or carries both of these mutations, and 2) the corresponding phenotypic presentation is stated as hepatic, neurologic, mixed or asymptomatic. Tables 3 and 4 present the number, age and nationality of patients that meet our inclusion criteria.

**Table 3:** Phenotypes of patients homozygous and/or heterozygous for the c. 2299insC reported in the literature.

Genotype	Exons	No. of patients (age years)	Phenotype	Nationality	References
c.2299insC/c.2299insC^a^	8/8	1 (7)	Hepatic	Greek	Panagiotakaki et al. [34]
		2 (NA)	Hepatic	Egyptian	Abdelghaffar et al. [35]
		1 (8)	Hepatic	Cypriot	Butler et al. [36]
		1 (6)	Hepatic	Iranian	Dastsooz et al. [37]
		1 (10)	Neurologic	Chinese	Gu et al. [38]
		2 (1.3)	Asymptomatic^b^	Italian	Nicastro et al. [18]
		1 (NA)	Asymptomatic^b^	Egyptian	Abdel Ghaffar et al. [19]
		2 (7.5)	Hepatic^c^	Lebanese	Our study
		1 (1)	Hepatic^d^	Lebanese	Our study
		2 (14)	Neurologic	Lebanese	Our study
p.Arg616Gln/c.2299insC	5/8	1 (34)	Hepatic	Yugoslavian	Loudianos et al. [39]
		1 (22)	Neurologic	Bulgarian	Mihaylova et al. [40]
		1 (21)	Mixed	Bulgarian	Mihaylova et al. [40]
p.Met645Arg/c.2299insC	6/8	1 (10)	Hepatic	Spanish	Margarit et al. [23]
c.2299insC/p.Arg816Ser	8/9	1 (26)	Neurologic	Austrian	Hofer et al. [41]
c.2299insC/p.Val949Gly	8/12	1 (26)	Neurologic	Brazilian	Deguti et al. [42]
c.2299insC/p.Ala1003Thr	8/13	1 (15)	Hepatic	Yugoslavian	Loudianos et al. [39]
		3(14)	Hepatic	Lebanese	Our study
		1 (15)	Asymptomatic	Lebanese	Our study
c.2299insC/p.His1069Gln	8/14	4 (23.75)	Neurologic	Yugoslavian	Loudianos et al. [39]
		1 (10)	Hepatic	Turkish	Simsek Papur et al. [43]
c.2299insC/3402delC	8/15	1 (33)	Neurologic	Yugoslavian	Loudianos et al. [39]
		1 (9)	Hepatic	Brazilian	Deguti et al. [42]
c.2299insC/p.Leu1255Ile	8/18	1 (10)	Hepatic	Korean	Yoo [2]
c.2299insC/NA^e^	8/NA	2 (21)	Mixed	Thai	Keandaungjuntr et al. [44]
		1 (NA)	Hepatic	Egyptian	Abdelghaffar et al. [35]
		1 (19)	Hepatic	Yugoslavian	Loudianos et al. [39]
		1 (10)	Hepatic	Brazilian	Deguti et al. [42]

a. The identified c.2298–2299insC is referred to as c.2299insC.

b. The majority of asymptomatic patients in this study had transaminitis or hepatomegaly.

c. Asymptomatic transaminitis.

d. Asymptomatic fatty liver.

e. Unidentified or not reported.

**Table 4:** Phenotypes of patients homozygous and/or heterozygous for the p. Ala1003Thr missense mutation reported in the literature.

Genotype	Exons	No. of patients (age years)	Phenotype	Nationality	References
p.Ala1003Thr/p.Ala1003Thr	13/13	1 (<8)	Hepatic	Indian	Kumar et al. [13]
c.2299insC/p. Ala1003Thr	8/13	1 (15)	Hepatic	Yugoslavian	Loudianos et al. [39]
		3 (14)	Hepatic	Lebanese	Our study
		1 (15)	Asymptomatic	Lebanese	Our study
p.Ala1003Thr/p.His1069Gln	13/14	1 (23)	Neurologic	Yugoslavian	Loudianos et al. [39]
		1 (26)	Neurologic	Danish	Moller et al. [30]
p.Ala1003Thr/p.Val1036Ile	13/14	1 (25)	Neurologic	Turkish	Simsek Papur et al. [43]
p.Ala1003Thr/NA^a^	13/NA	1 (16)	Neurologic	Yugoslavian	Loudianos et al. [39]
		1 (20)	Neurologic	Greek	Butler et al. [36]

A total of 14 patients, including 5 of ours (this study), were homozygous for the c. 2299insC mutation (Table 3). The most common presentation was hepatic identified in 57%, while each of neurologic and asymptomatic equally presented at 21.4%. The majority of asymptomatic patients had transaminitis [18], [19].

Twenty four compound heterozygous patients, carrying the c. 2299insC/allele, were identified worldwide with 12 hepatic (50%), 8 neurologic (33.3%), 3 mixed (12.5%) phenotypes and 1 asymptomatic (4%).

There were 11 patients carrying the p. Ala1003Thr mutation (c.3007G>A substitution), one of whom was homozygous and presented with hepatic phenotype at a young age (Table 4). Ten patients were compound heterozygous carrying the p. Ala1003Thr mutation on one allele. Patients with c. 2299insC on the second allele had a predominant hepatic presentation (4/5) with mean age of 13.5 yrs, whereas all patients (5/5) with non c. 2299insC on the second allele had neurologic presentation with a mean age of 22 years. One asymptomatic patient with the (c. 2299insC/p. Ala1003Thr) was diagnosed by screening at 15 years of age.

## Discussion

We report in this paper the genotype-phenotype correlations in a very large single family with extensive consanguinity and a high prevalence of WD. The main finding of our study and of the literature review is an association between the c. 2299insC mutation in the homozygous and the compound heterozygous state and hepatic phenotype. On the other hand, there is an association with the mutation p. Ala1003Thr in the compound heterozygous state with a neurologic phenotype. Based on the most recent EASL practice guidelines, all patients in our study have definite Wilson disease [20].

More than 500 mutations have been described in WD, and missense ones are the most common. The majority of those mutations are rare and most patients are compound heterozygote. Hence, establishment of convincing genotype correlations has been difficult and results inconclusive [8], [21]–[25]. In addition, other epigenetic and environmental factors play a role in disease presentation. Thus, conducting large consanguineous family studies in homogenous populations would facilitate establishment of genotype phenotype correlations as members of those families are more likely to share the same genetic and environmental factors and to be homozygous [9], [26]. Finally, high concordance of the clinical and biochemical manifestations of WD among siblings suggests that members of the same family may have similar phenotypes [6], and that the influence of other genetic, epigenetic, and environmental factors may be similar in those members. As shown in the pedigree, 7 of our patients belonged to 3 nuclear sub-families reflecting the extensive consanguinity.

This is the largest single family ever reported with such a high prevalence of WD and of carriers of *ATP7B* mutations. All members of the family belong to the same village and ethnic group, with many members sharing a common household. Hence they had common environmental and dietary habits. Furthermore, consanguineous marriage is the norm in this family as can be seen in the pedigree (Fig. 1). In addition, the 9 patients with WD are being taken care of in single tertiary care center, and thus all diagnostic procedures are uniformly standardized.

Six of our patients had liver disease, and three of those had full blown cirrhosis and portal hypertension. No liver biopsy was performed, but the diagnosis of liver cirrhosis was firmly established based on clinical, laboratory and imaging grounds as suggested elsewhere [27], [28] . All six patients had the c. 2299insC mutation in the homozygous or compound heterozygous state, and 3 of them were females. However, two male patients that were homozygous for the c. 2299insC mutation had neurologic manifestations of Wilson disease, and one of them developed cirrhosis and portal hypertension years later. No liver biopsy was done on either.

As seen in table 3, there were 38 patients carrying the c. 2299insC mutation in the homozygous or compound heterozygous state worldwide. The frequencies of the hepatic, neurologic, mixed and asymptomatic phenotype was 53%, 29%, 11% and 8%, respectively. Four of the patients who had neurologic phenotype were compound heterozygous for p. His1069Gln which is known to be associated with neurologic phenotype [29]. Conversely, there were 10 compound heterozygous patients carrying the p. Ala1003Thr mutation (Table 4). For those carrying the c. 2299insC as the second mutation, hepatic phenotype was predominant, while for all others (5 out of 5), neurologic phenotype was predominant. Thus, an important question arises in compound heterozygous patients: which mutation dictates the phenotype of the patient? In view of the small number of patients and large number of mutations, this question may be difficult to answer. A suggestion by MØller LB et al [30] was made that the milder mutation dictates the age of onset and possibly the phenotype. She considered both c. 2299insC and p. His1069Gln to be severe mutations based on earlier onset of symptoms. In our study, the mean age of patients carrying the c. 2299insC mutation is 14.4 years and of those carrying the p. Ala1003Thr mutation is 17 years. Based on that, both mutations would be considered “severe”. However, extensive heterogeneity exists in age of onset as shown in Tables 3 and 4.

In an ongoing study on genotype-phenotype correlations in Caucasian patients, preliminary results suggest that hepatic phenotype is present in 69% of patients who are compound heterozygous c.2299 insC/p. His1069Gln [8]. This is consistent with our suggestion that c. 2299insC is associated with hepatic phenotype, even when it is combined with another mutation that is associated with neurologic phenotype.

What then could explain the severe hepatic phenotype of a patient who is homozygous for p. Ala1003Thr as reported by Kumar et al [13]? We had reported before a strong association between hepatic phenotype, hepatic failure and homozygosity for missense mutations other than p. His1069Gln [12] . Thus, it looks like there are multiple genetic determinants of phenotype in WD, including homozygosity, the type of mutation and its severity, the weight of individual mutations in those who are compound heterozygote, as well as other known and unknown genetic and epigenetic factors.

Establishing genotype–phenotype correlations is clearly important for appropriate patient management, for initiation of early therapy in asymptomatic patients to prevent certain complications, and for monitoring the efficacy of treatment. Furthermore, it enhances understanding of the molecular pathogenesis of the disease. However, it is still fraught with extensive difficulties. In addition to the complex genetic factors, the extensive phenotypic heterogeneity of WD, and the small number of patients, other factors seem to be important in determining phenotype in WD. These include sex, ethnicity, environmental and dietary factors as well as percentage residual activity of the translated protein. Furthermore, younger age of onset of symptoms may contribute to the phenotype as well.

In the absence of a purified protein, our current understanding of how mutations and SNPs affect protein function, and therefore phenotype, remains speculative. It is plausible to assume that derangement in protein function varies with nature and position of mutation. Discordance in monozygotic twins suggests that environmental, dietary, and epigenetic factors including copper chaperones may contribute to the phenotype [31]. In addition, the expression of the *ATP7B* gene product and/or its isoforms is another factor [32]. Alternative splicing of the ATP7B gene is tissue specific. Whereas all exons are expressed in the translated liver protein, several alternatively spliced isoforms are expressed in the brain; resulting from skipping certain exons [33]. Thus translation of the *ATP7B* gene, carrying a mutation in an alternatively spliced exon, may have no phenotypic effect or manifest clinical dysfunction of that organ. Though difficult to perform *in vivo*, variation in the expression level of the different *ATP7B* isoforms in liver and brain might help in explaining the phenotypic diversity in subjects with identical genotype.

Our study has several strengths. The family we evaluated is very large and has extensive consanguinity. Its members belong to the same ethnic group and they have the same environmental exposure and similar dietary habits. Thus, potentially compounding factors on the effect of genotype on phenotype are eliminated or reduced. There are also limitations to our study. Twelve members of this family died apparently of WD. We have no way of ascertaining their phenotype. In addition, individuals belonging to such a poor family may not seek medical attention if they develop mild symptoms, due to financial limitations. Furthermore manifestations of mild phenotypes may not be easily recognized. Finally, we depended in our literature review on cases where the phenotypes are clearly indicated.

In conclusion, phenotype-genotype correlations in WD patients remain a challenge as long as diagnosis at the asymptomatic stage is not possible and functional assay of purified normal and mutant protein is still unavailable.

## Supporting Information

Table S1 **Normal (N) and mutant (M) nucleotide probes of mutations and SNPs identified in the S- Family in Exons: 8, 10, 12, 13, and 16.**
(DOCX)
