# Supplementary material for: Phenotype-Genotype Correlation in Wilson Disease in a Large Lebanese Family: Association of c.2299insC with Hepatic and of p. Ala1003Thr with Neurologic Phenotype
Source: PLoS One. 2014 Nov 12;9(11):e109727. doi: 10.1371/journal.pone.0109727 (PMC4229086; doi:10.1371/journal.pone.0109727)
Supplement: Table S1 — Normal (N) and mutant (M) nucleotide probes of mutations and SNPs identified in the S- Family in Exons: 8, 10, 12, 13, and 16. (DOCX) [file pone.0109727.s001.docx]

**Table S 1**

**Normal and Mutant probes**

| Exon 8-N | 5'-GACACGCCCCCCATGCTCTTT-3' | Exon8-M | 5'GACACGCCCCCC**C**ATGCTCTTT-3' |
| --- | --- | --- | --- |
| Exon10-N | 5'-GATATCGTCA**A**GGTGGTCCCT-3' | Exon10-M | 5'-GATATCGTCA**G**GGTGGTCCCT-3' |
| Exon12-N | 5'-TTGGTGTTGTTCAGA**A**A-3' | Exon12-M | 5'-TTGGTGTTGTTCAGA**G**A-3' |
| Exon13-N | 5'-GGGGTGGCC**G**CGCAGAACGGC-3' | Exon13-M | 5'-GGGGTGGCC**A**CGCAGAACGGC-3' |
| Exon16-N | 5'-GATGCAG**T**CCCCCAGACC-3' | Exon16-M | 5'-GATGCAG**C**CCCCCAGACC-3' |

**Table S1 legend:**

Normal (N) and mutant (M) nucleotide probes of mutations and SNPs identified in the S- Family in Exons : 8, 10, 12, 13, and 16.
